# Multi-modal contrastive learning of subcellular organization using DICE

**DOI:** 10.1093/bioinformatics/btae387

**Published:** 2024-09-04

**Authors:** Rami Nasser, Leah V Schaffer, Trey Ideker, Roded Sharan

**Affiliations:** School of Computer Science, Tel Aviv University, Tel Aviv 69978, Israel; Department of Medicine, University of California, San Diego, San Diego, CA 92037, United States; Department of Medicine, University of California, San Diego, San Diego, CA 92037, United States; Department of Computer Science and Engineering, University of California, San Diego, San Diego, CA 92037, United States; Moores Cancer Center, University of California, San Diego, San Diego, CA 92037, United States; Department of Bioengineering, University of California, San Diego, San Diego, CA 92037, United States; School of Computer Science, Tel Aviv University, Tel Aviv 69978, Israel

## Abstract

**Summary:**

The data deluge in biology calls for computational approaches that can integrate multiple datasets of different types to build a holistic view of biological processes or structures of interest. An emerging paradigm in this domain is the unsupervised learning of data embeddings that can be used for downstream clustering and classification tasks. While such approaches for integrating data of similar types are becoming common, there is scarcer work on consolidating different data modalities such as network and image information. Here, we introduce DICE (Data Integration through Contrastive Embedding), a contrastive learning model for multi-modal data integration. We apply this model to study the subcellular organization of proteins by integrating protein–protein interaction data and protein image data measured in HEK293 cells. We demonstrate the advantage of data integration over any single modality and show that our framework outperforms previous integration approaches.

**Availability:**

https://github.com/raminass/protein-contrastive

**Contact:**

raminass@gmail.com

## 1 Introduction

Integrative modeling of biological data is becoming a central paradigm in computational biology as in other research domains. Each data type provides a different view of the question at hand, enhancing the predictive power of a joint model. For example, the integration of different biological networks has been shown to benefit gene functional annotation and module detection (Malod-Dognin *et al.* 2019, Forster *et al.* 2022, Nasser and Sharan 2023).

The integration problem becomes potentially harder when the input data types have different representations, referred to as *data modalities* below. Depending on the experimental framework used to generate the data, a protein may be represented as a sequence of amino acids, a list of 3D structural coordinates, a set of interactors or an image of cellular locations. This variety calls for learning a joint representation in which the different modalities are projected to a joint Euclidean space to allow cross-modality comparison, imputation, and integration.

One of the prime examples for multi-modal integration outside the biological domain is the integration of images and their captions using contrastive learning (Radford *et al.* 2021). The main idea behind this method, called CLIP (contrastive language-image pre-training), is to learn a joint representation in which matching image-caption pairs are required to map to close-by locations while nonmatching pairs are required to map to far away locations. Integration of different data modalities through embedding is starting to gain momentum also in the biological domain. For example, the Multi-Scale Integrated Cell (MuSIC) approach (Qin *et al.* 2021) uses a supervised integration scheme to learn similarities between proteins based on their location and interaction data. These similarities are then employed to construct a hierarchical map of subcellular components. A state-of-the-art approach for the integration problem, called MUSE (Bao *et al.* 2022) (multi-modal structured embedding), co-embeds transcriptomics and imaging data by characterizing clustering structures in each modality and using a self-reconstruction loss.

Here, we borrow ideas from CLIP to design a multi-modal biological Data Integration through Contrastive Embedding (DICE) framework. DICE receives two data modalities as input and learns a joint embedding that can be used for downstream prediction tasks. We demonstrate our framework by integrating protein image and interaction data from HEK293 cells to learn a hierarchical map of subcellular components. We compare DICE to previous approaches for the integration problem, showing the advantage of a joint embedding in general, and our framework in particular, in producing solutions that agree well with current functional annotation of proteins.

## 2 Materials and methods

We start with a general formulation of the problem and then derive a focused formulation that is suited to the application at hand. We assume we are given two data modalities describing protein-level properties. We further make a simplifying assumption that each modality has a corresponding encoder that can encode any sample into a vector of real numbers. Our goal is to learn a joint embedding of the two modalities so that any pair of cross-modality vectors describing the same protein will map close to one another, while any pair of vectors describing different proteins map to far away locations. As in recent contrastive learning models (Bachman *et al.* 2019, Chen *et al.* 2020a, Radford *et al.* 2021, Zhang *et al.* 2022), our DICE approach learns representations by maximizing agreement between different modality views of the same protein via a contrastive loss in the latent space. In the following subsections we describe our method’s components in detail.

### 2.1 Problem formulation

DICE receives as input two data modalities that describe the same *n* proteins: $V_{n\times f}$ and $X_{n\times k}$. The goal is to embed $V,X\underset{g_{v},g_{x}}{\to }Z_{v},Z_{x}$ into a shared latent space $Z\in R^{l}$, where *g_v_* and *g_x_* are parameterized projection modules. A leading approach to learn this joint embedding is a self-supervised contrastive learning approach (Chen *et al.* 2020a, Radford *et al.* 2021, Zhang *et al.* 2022). This approach is based on maximizing the similarity of the embeddings that correspond to the same element, while minimizing embeddings that correspond to different elements. We follow this approach and define the goal of the embedding as the minimization of a contrastive loss that is defined as the sum of the following loss terms:


(1) $$L_{v2x}=-\sum_{i=1}^{n}\;\text{log}\;\frac{\;\text{exp}\;\left(\tau \mathrm{sim}(v_{i},x_{i})\right)}{\sum_{j\in n}\;\text{exp}\;\left(\tau \mathrm{sim}(v_{i},x_{j})\right)+\sum_{j\neq i}\;\text{exp}\;\left(\tau \mathrm{sim}(v_{i},v_{j})\right)}$$

(2) $$L_{x2v}=-\sum_{i=1}^{n}\;\text{log}\;\frac{\;\text{exp}\;\left(\tau \mathrm{sim}(x_{i},v_{i})\right)}{\sum_{j\in n}\;\text{exp}\;\left(\tau \mathrm{sim}(x_{i},v_{j})\right)+\sum_{j\neq i}\;\text{exp}\;\left(\tau \mathrm{sim}(x_{i},x_{j})\right)}$$

where *τ* is a scale parameter and sim() is the cosine similarity measure. Notably, this loss function accounts for both intra-modality and cross-modality pairs, unlike CLIP which accounts for cross-modality pairs only.

For both *g_v_* and *g_x_* we use a simple multi-linear perceptron (MLP) with one hidden layer. Thus, the corresponding projections are: $z_{v_{n}}=g_{v}(v_{n})=W_{v}^{\left(2\right)}\sigma (W_{v}^{\left(1\right)}v_{n})$ and $z_{x_{n}}=g_{x}(x_{n})=W_{x}^{\left(2\right)}\sigma (W_{x}^{\left(1\right)}x_{n})$, where *σ* is a ReLU activation function.

### 2.2 Application to subcellular organization of proteins

We focus on the problem of subcellular organization of proteins based on global (large-scale) localization images and local (small-scale) interaction information. We integrate immunofluorescence images from the Human Protein Atlas (Thul *et al.* 2017), with protein–protein interactions from the Bioplex network (Huttlin *et al.* 2021) in HEK293T cells. The network has 14 032 proteins and 127 732 interactions. The image data contains 2341 immunofluorescence images, with 2–6 images per protein. The network and the image data share 876 common proteins.

For image encoding we use DenseNet (Ouyang *et al.* 2019), a convolutional neural network pre-trained to classify Human Protein Atlas images (Thul *et al.* 2017). For encoding the network, we use the well-known node2vec (Grover and Leskovec 2016). We opt for these encodings for fair comparison to the previous works that used them.

During the learning process, each time we randomly sample a mini-batch of *B* matching (*v_b_*, *x_b_*) pairs. We train the model to predict the matching *B* pairs out of all possible pairs, aiming to maximize the similarity between $\left(z_{v_{b}},z_{x_{b}}\right)$ of true pairs and minimize the similarity between all other $3(B^{2}-B)$ pairs. To this end, we use the cross-entropy loss function described above, where *n* is replaced by *B*.

After learning, in order to construct a representative embedding for each protein that accounts for its image-based and network-based embeddings, we fuse those embeddings into a joint representation. While there are many potential fusion functions (Lahat *et al.* 2015), we simply use concatenation: $Z=[g_{v}(V)|g_{x}(X)]$.

### 2.3 Implementation and run-time details

All reported runs were performed in Ubuntu 20.04.6 LTS using a 1-core CPU (x86_64). Code is available at https://github.com/raminass/protein-contrastive. The training time of an epoch is ∼2 s, where we used a batch size of 128 and trained for 100 epochs.

### 2.4 Performance evaluation

We evaluated our method using the recent benchmark of BIONIC Forster *et al.* (2022) with the following tasks: (i) protein module detection; and (ii) supervised protein function prediction. While the former task is evaluated in BIONIC through hierarchical clustering, we chose for efficiency to employ instead the state-of-the-art Louvain clustering algorithm (Blondel *et al.* 2008). Module benchmarks in human were derived from KEGG pathways (Kanehisa and Goto 2000) (excluding metabolic pathways), GO cellular components (Ashburner *et al.* 2000) and CORUM complexes (Giurgiu *et al.* 2019).

## 3 Results

We devised a contrastive learning approach to integrate multi-modal data for subcellular mapping of proteins. Our method, called DICE, learns to embed the two modalities in a joint space where samples that correspond to the same protein map to close-by locations while other pairs map to far-away locations. The resulting embeddings are fused to produce a joint representation of proteins that can be used for downstream tasks such as cellular component mapping and protein function prediction.

We applied our method to jointly embed network and image information on 876 human proteins as measured in HEK293 cells. In order to evaluate the quality of the embeddings generated by our proposed method, we followed the BIONIC pipeline (Forster *et al.* 2022) which quantifies the quality of protein embeddings in terms of their utility in module detection and function prediction tasks (Section 2). For evaluation, we used three module standards: KEGG (Kanehisa and Goto 2000) (excluding metabolic pathways), GO cellular component (Ashburner *et al.* 2000), and CORUM (Giurgiu *et al.* 2019).

**Figure 1.**
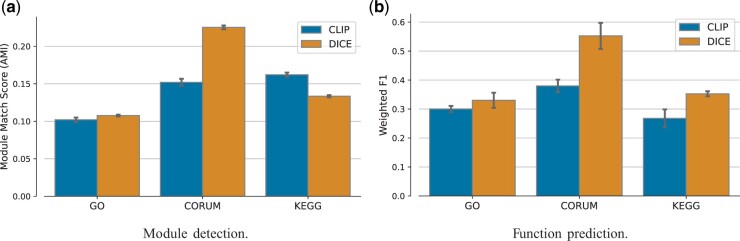
Performance evaluation of the learned joint representation between vanilla CLIP and DICE. (a) Module detection performance using the adjusted mutual information (AMI) measure. (b) Function prediction performance evaluated by Weighted F1 which is the average per-class F1 score (harmonic mean of precision and recall).

A key consideration in contrastive learning is the design of the loss function (Chen *et al.* 2020a,b, Radford *et al.* 2021) and specifically the choice of negative examples (Wang and Isola 2020). Empirical research has consistently demonstrated that an increase in the number of negative samples correlates with improved performance in downstream tasks (Chen *et al.* 2020a, He *et al.* 2020, Tian *et al.* 2020), which explains the large batch size in CLIP (32 768). In our specific setting, where there are only a total of 876 proteins, this guideline is difficult to satisfy. To combat data sparsity, we added intra-modal pairs as negatives to the CLIP loss function, which focuses on inter-modal pairs. Comparing our method’s extended loss to the vanilla CLIP loss, we observed that it leads to improved embeddings with respect to all but one evaluation tasks (Fig. 1).

**Figure 2.**
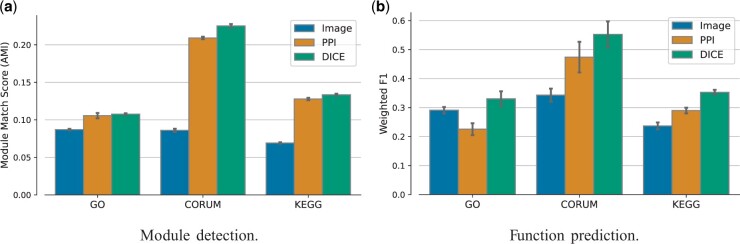
Performance evaluation of the learned joint representation against the individual modalities. (a) Module detection performance using the adjusted mutual information (AMI) measure. (b) Function prediction performance evaluated by Weighted F1 which is the average per-class F1 score.

Next, we evaluated our embedding against the input embeddings of each of the individual modalities. The results are shown in Fig. 2 and demonstrate the superiority of the integrated representation across the three standards and the two prediction tasks.

**Figure 3.**
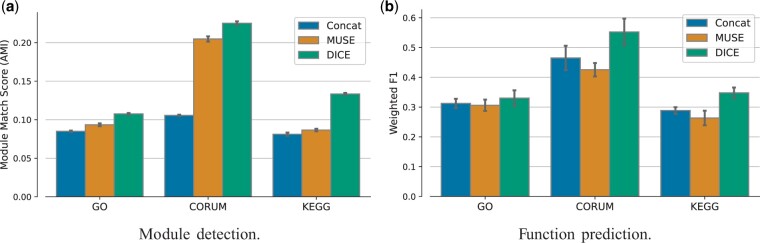
Performance evaluation of DICE against previous fusion methods. (a) Module detection performance using the adjusted mutual information (AMI) measure. (b) Function prediction performance evaluated by Weighted F1 which is the average per-class F1 score.

After showing the utility of integration, we turn to compare DICE to previous integrative approaches. As a benchmark, we use a layman method (Concat) that concatenates the features of the individual modality embeddings. This is the first (unsupervised) step in the more involved supervised integration approach employed in MuSIC (Qin *et al.* 2021) as well as in the classification method of Wang *et al.* (2022). In addition, we compare DICE to MUSE (Bao *et al.* 2022), a co-embedding approach that assumes that the data has a well-defined cluster structure. The results are summarized in Fig. 3 and show that our method dominates the other methods and produces embeddings that agree better with the known standards.

**Figure 4.**
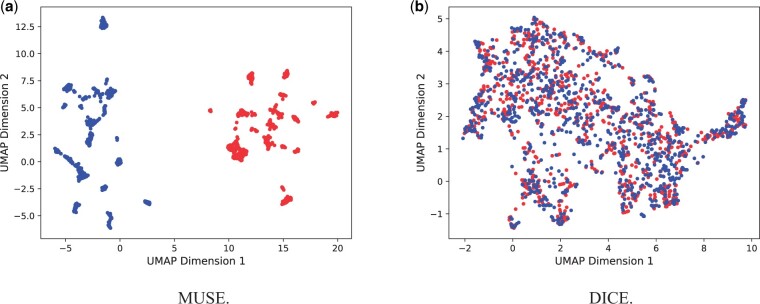
UMAP projections of latent network-based (red) and image-based (blue) protein embeddings from MUSE (a) and DICE (b).

Key properties of contrastive learning are the alignment of matching pairs that is explicitly modeled by the loss function and the uniformity of nonmatching pairs (Wang and Isola 2020). To examine the alignment and uniformity of our embedding, we visualized it using the UMAP dimensionality reduction algorithm (McInnes *et al.* 2018). As shown in Fig. 4, both modalities are well aligned for each protein (mean distance of 0.69) and are well distributed over the plane. In contrast, the two modalities are completely separated in the MUSE embedding (mean distance of 9.57 between matching pairs, significantly larger than for DICE with a Mann-Whitney *p *=* *0).

**Figure 5.**
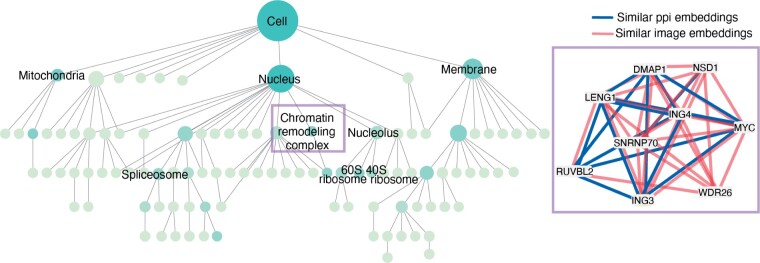
Hierarchy of protein assemblies constructed using the DICE embeddings. Nodes represent protein assemblies and edges represent hierarchical containment. Nodes are shaded based upon overlap with a Gene Ontology cellular component term. Right panel contains a chromatin remodeling assembly subnetwork, where edges represent high similarity between the original image-based (red) or PPI-based (blue) protein embeddings (top 10% most similar pairs).

As an application of the DICE embeddings, we performed clustering on the similarities between embeddings at multiple resolutions to construct a hierarchy of subcellular systems Zheng *et al.* (2021). The final hierarchy (Fig. 5) contains 110 protein assemblies. Out of the 47 assemblies with 10 or more proteins, 22 had significant overlap (FDR <5%) with a Gene Ontology Cellular Component term, recovering known assemblies across scales including the nucleus, mitochondria, spliceosome, and ribosomal subunits. One protein assembly revealed by the DICE embeddings, a chromatin remodeling assembly, contained protein pairs with similar original embeddings from both modalities (top 10% most similar pairs in the original modality). This assembly includes proteins that participate in the H4 histone acetyltransferase complex (ING3, RUVBL2, DMAP1), as well as other chromatin binding proteins informed by PPI data, imaging data, or both. This application highlights how integration with DICE enables elucidation of protein assemblies informed by different original data modalities.

**Figure 6.**
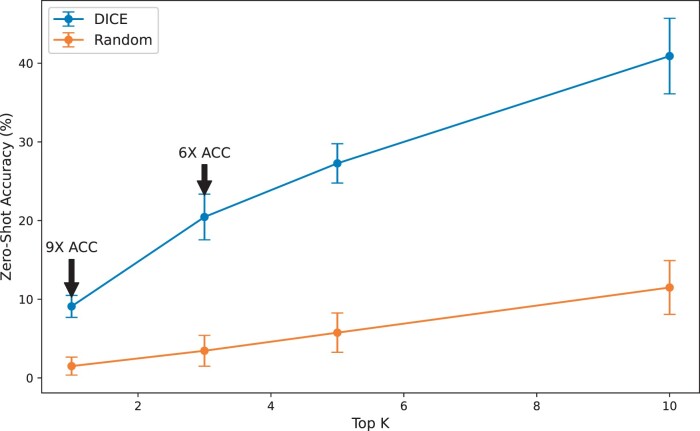
Evaluation of zero-shot classification accuracy compared to a random baseline across different Top-K prediction scenarios. Error bars denote the 95% confidence interval for each Top-K accuracy point. DICE dominates the random baseline, with a clear margin of improvement as indicated by the 6× and 9× marks at Top-3 and Top-1, respectively.

Last, we assessed the capabilities of a contrastive learning model within a zero-shot learning framework (Lampert *et al.* 2013, Lei Ba *et al.* 2015) to match images to proteins that were not present during the training phase. We implemented a 10-fold cross-validation strategy, where the model is trained on 9-folds and the matching pairs in the tenth fold are used for testing. For simplicity, after training, each image in the test set is assigned to one of its most similar proteins where similarity is measured by cosine similarity of the corresponding embeddings. Figure 6 illustrates the model’s assignment accuracy compared to a random baseline across Top-1, Top-3, Top-5, and Top-10 prediction scenarios. The superior performance of DICE over the random baseline is evident, particularly at Top-1 and Top-3 scenarios, where it achieved a 9-fold and 6-fold increase in accuracy, respectively. These results highlight the ability of zero-shot learning using DICE to generalize from seen to unseen classes.

## 4 Conclusions

We developed a novel contrastive learning framework for integrating multi-modal data. We applied this framework to protein image and interaction data for learning the subcellular organization of proteins. Due to data scarcity, we modified a previous loss function that focused on inter-modal pairs to include also the separation of intra-modal pairs. We demonstrated that the resulting embedding is effective in protein module detection and function prediction, outperforming the single modalities as well as previous multi-modal integration approaches.

We also tested the utility of our framework in classifying unseen objects. The contrastive learning framework, by capturing the nuanced relationships between images and nodes, demonstrates a powerful capacity for zero-shot classification, with application to cases where direct knowledge of specific classes is not available during training.

The generation of a data-derived hierarchy of subcellular components enables revealing assemblies of proteins that are not biased toward well-studied proteins in literature-curated resources such as the Gene Ontology. Here, we focused on the subset of proteins in both the imaging and interaction datasets, which we found previously covers a similar distribution of subcellular locations as all human proteins (Qin *et al.* 2021). Important areas for future models will be to expand to additional data modalities and to include proteins only present in subsets of the data, which will result in broader maps of cell structure that cover more protein assemblies.

While the subcellular map we have built was based on protein location and interaction data, additional modalities such as protein sequence and structure information may inform subcellular organization. For example, hydrophobic regions in inner membrane proteins enable their integration into lipid bilayers, while nuclear localization signals, which are specific amino acid sequences, direct proteins to the nucleus. Generalizing our approach to more than two modalities could lead to ever more accurate maps of the cell.

## Supplementary Material

btae387_Supplementary_Data
